# Myocardial injury: where inflammation and autophagy meet

**DOI:** 10.1093/burnst/tkac062

**Published:** 2023-03-01

**Authors:** Chunping Liu, Yanjiao Liu, Huiqi Chen, Xiaofei Yang, Chuanjian Lu, Lei Wang, Jiahong Lu

**Affiliations:** State Key Laboratory of Quality Research in Chinese Medicine, Institute of Chinese Medical Sciences, University of Macau, Macau, China; State Key Laboratory of Dampness Syndrome of Chinese Medicine, The Second Affiliated Hospital of Guangzhou University of Chinese Medicine, Guangzhou, 51080, China; Guangdong-Hong Kong-Macau Joint Lab on Chinese Medicine and Immune Disease Research, Guangzhou, 510080, China; State Key Laboratory of Dampness Syndrome of Chinese Medicine, The Second Affiliated Hospital of Guangzhou University of Chinese Medicine, Guangzhou, 51080, China; State Key Laboratory of Dampness Syndrome of Chinese Medicine, The Second Affiliated Hospital of Guangzhou University of Chinese Medicine, Guangzhou, 51080, China; State Key Laboratory of Dampness Syndrome of Chinese Medicine, The Second Affiliated Hospital of Guangzhou University of Chinese Medicine, Guangzhou, 51080, China; State Key Laboratory of Dampness Syndrome of Chinese Medicine, The Second Affiliated Hospital of Guangzhou University of Chinese Medicine, Guangzhou, 51080, China; Guangdong-Hong Kong-Macau Joint Lab on Chinese Medicine and Immune Disease Research, Guangzhou, 510080, China; State Key Laboratory of Dampness Syndrome of Chinese Medicine, The Second Affiliated Hospital of Guangzhou University of Chinese Medicine, Guangzhou, 51080, China; State Key Laboratory of Quality Research in Chinese Medicine, Institute of Chinese Medical Sciences, University of Macau, Macau, China

**Keywords:** Autophagy, Inflammation, Myocardial injury

## Abstract

Autophagy is a highly conserved bulk degradation mechanism that degrades damaged organelles, aged proteins and intracellular contents to maintain the homeostasis of the intracellular microenvironment. Activation of autophagy can be observed during myocardial injury, during which inflammatory responses are strongly triggered. Autophagy can inhibit the inflammatory response and regulate the inflammatory microenvironment by removing invading pathogens and damaged mitochondria. In addition, autophagy may enhance the clearance of apoptotic and necrotic cells to promote the repair of damaged tissue. In this paper, we briefly review the role of autophagy in different cell types in the inflammatory microenvironment of myocardial injury and discuss the molecular mechanism of autophagy in regulating the inflammatory response in a series of myocardial injury conditions, including myocardial ischemia, ischemia/reperfusion injury and sepsis cardiomyopathy.

Highlights

- Briefly reviews the crosstalk between autophagy and inflammation in myocardial injury.
- Discusses the molecular mechanism of autophagy in regulating the inflammatory response in different cell types of myocardial injury.
- Discusses the prospect of autophagy in the treatment of myocardial injury and provides some guidance for the clinical treatment of myocardial injury.

## Background

Cardiovascular disease (CVD) is one of the most important causes of human death worldwide. According to the latest data, the number of CVD deaths per year is estimated to be 18.6 million [1,2]. Ischemic heart disease can be classified as coronary artery disease and myocardial disease. The deterioration of ischemic myocardium is the main cause of myocardial injury. A large number of cardiac muscle cells die during ischemia and are eventually replaced by noncontractible scar tissue, which results in heart failure [3]. Myocardial infarction (MI) is a serious disease that endangers human health and ranks as the leading cause of death worldwide [4]. With the extensive development of reperfusion therapy, the mortality rate of early MI has been significantly reduced [5]. However, the incidence of events such as heart failure and sudden death caused by negative ventricular remodeling remains high, representing the main factor restricting the long-term prognosis of MI patients [4]. In addition to myocardial ischemia/reperfusion (I/R) injury, diabetic cardiomyopathy, chemotherapy-related cardiomyopathy, sepsis cardiac dysfunction and dilated cardiomyopathy can all cause serious myocardial injury [6–9]. Cardiovascular diseases pose a serious threat to human health and also cause heavy social and economic burdens worldwide. Therefore, it is very important to explore new repair strategies for myocardial injury.

Inflammation is a protective response to tissue damage that is mainly mediated by pattern recognition receptors and the identification of pathogen-associated molecular patterns or damage-associated molecular patterns, which are conserved structural features recognized by the innate immune system. The activation of the innate immune system subsequently causes increased activation of protein signal transduction cascades and activates the adaptive immune response [5,10]. Approximately 2–4 billion cardiomyocytes are localized in adult ventricles; these cells are in a state of terminal differentiation and lack the ability to re-enter the cell cycle and proliferate. Serious myocardial injury leads to irreversible cardiac remodeling and dysfunction. The occurrence, development and repair of inflammation after myocardial injury all involve a variety of immune cells, which produce a large number of inflammatory cytokines and drive different inflammatory cells to infiltrate inflammatory tissue to ultimately improve cardiac inflammation and fibrosis [11]. Therefore, only by fully understanding the mechanism of inflammation and repair caused by myocardial injury can we find a better method to improve it. The inflammatory response is the core pathological mechanism of acute myocardial injury. Methods for effectively improving the microenvironment that occurs during myocardial inflammation and promoting the repair of damaged blood vessels or myocardium have become key to the improvement of the clinical efficacy of CVD treatments.

Autophagy refers to the formation of autophagosomes surrounded by double membranes, and this process is induced by various conditions, such as inflammation or starvation. Autophagosomes and lysosomes fuse to degrade autophagic substrates, which is a self-protective mechanism to maintain the homeostasis of the intracellular environment [12,13]. Recent studies have shown that a variety of innate immune recognition receptors can activate autophagy while inducing inflammatory responses, and autophagy has a significant positive/negative regulatory effect on inflammatory responses [14,15]. Autophagy defects or autophagy-related gene (ATG) mutations are closely related to the occurrence of inflammatory diseases. Autophagy plays an important role in the positive regulation of the inflammatory response to myocardial injury by clearing inflammasomes, cytokines and cellular components. In addition, autophagy can weaken the inflammatory response by promoting the elimination of apoptotic cells and damaged mitochondria. Autophagy plays both positive and negative regulatory roles in the process of myocardial injury [16]. Maintaining autophagy homeostasis may represent an important strategy to alleviate myocardial inflammation.

In conclusion, the inflammatory response is the key pathological mechanism of myocardial ischemia, I/R injury, septic cardiomyopathy and diabetic cardiomyopathy. Studies have shown that autophagy plays an important role in maintaining myocardial microenvironment homeostasis by regulating the inflammatory response. Here, we review the progress made by recent research on the crosstalk between autophagy and inflammation and discuss their roles in myocardial injury, aiming to provide new insight into regulating the inflammatory microenvironment in myocardial injuries.

## Review

### Molecular mechanism of autophagy

**Figure 1:**
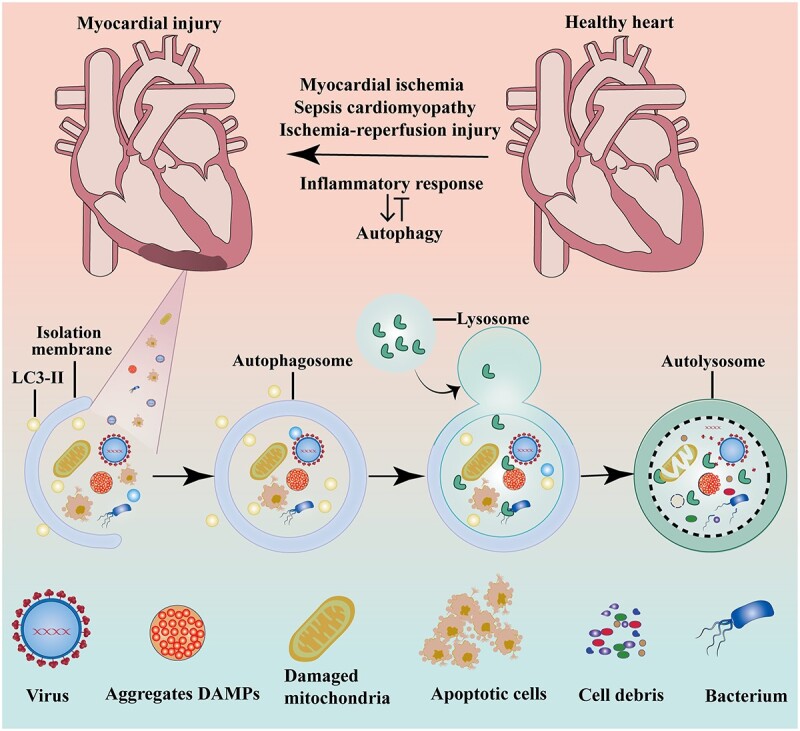
The role of autophagy in myocardial damage. *LC3* Microtubule-associated protein 1 light chain 3, *DAMPs* damage-associated molecular patterns

Autophagy is the self-clearing mechanism of the body, whereby damaged organelles and biological macromolecules are degraded to maintain cell homeostasis for the recycling of cell energy and substances [17]. Autophagy can be activated by starvation, hypoxia, reactive oxygen species (ROS) and mitochondrial damage. Cells can eliminate damaged or harmful components and protect their survival during stimulation by a variety of stressors through autophagy [18] **(**Figure 1**)**. Autophagy mainly includes three types: macroautophagy, microautophagy and chaperone-mediated autophagy. Generally, the word ‘autophagy’ refers to macroautophagy and the most important feature of autophagy is the formation of autophagosomes [19]. In addition to classic autophagy, microtubule-associated protein 1 light chain 3 (LC3)-associated phagocytosis (LAP) [20] is an important nonclassical pathway for the degradation of extracellular contents, including bacteria, dead cells and abnormal extracellular protein aggregates. LAP is triggered after phagocytic particles bind to cell surface receptors, which results in the recruitment of the autophagy machinery to phagosome-containing stimuli and promotes rapid phagosome maturation and the degradation of phagocytic pathogens.

A major breakthrough in the scientific understanding of autophagy involved the discovery of >40 ATGs in yeast, and most of these genes have mammalian homologs that jointly participate in the regulation of the initiation, extension and termination of autophagy and its degradation mechanism [21]. Among the >40 ATGs identified in yeast, 15 are known as core ATGs that are required for nonselective and selective autophagy [22]. In yeast models and higher eukaryotes, autophagy activity mostly occurs in the form of functional clusters. The ULK1 serine threonine kinase complex (including ULK1, FIP200, ATG13 and ATG101) plays an important role in the initiation of autophagy [23], which is regulated by mammalian target of rapamycin complex 1 (mTORC1). mTORC1 binds to the Atg1/ULK1 complex and phosphorylates ULK1- and ATG13-specific sites, inhibiting the autophagy-promoting kinase activity of the ULK1 complex and the initiation of autophagy. However, under stress conditions, such as tissue injury, the mTORC1 and ULK1 complexes are separated and the phosphorylation of ATG13 and ULK1 at specific sites is reversed. The ULK1 complex is activated under the action of auto-phosphorylation and further phosphorylates ATG13 and other proteins and targets the assembly of the proautophagosome, thus initiating autophagy. In addition, ATG9 and the VPS34 complex are also involved in regulating the formation of autophagosomes [24]. Several core ATGs function in a shared process that has some similarities to autophagy but involves digestion of extracellular material. In the process known as LAP, single membranes of large endocytic vacuoles phagocytize extracellular cargo, are decorated by lipidation of LC3 and transfer to lysosomes for degradation [20]. Significant gaps remain in the ability to distinguish LAP from typical autophagy in terms of molecular mechanisms and specificity.

### Inflammation in myocardial injury

Inflammation is the complex biological response of body tissues to noxious stimuli, such as pathogens, damaged cells or irritants, and it involves the protective responses of immune cells, blood vessels and molecular mediators [25]. Inflammation leads to the elimination of necrotic cells and tissues damaged during injury and initiates tissue repair [26].

More than 70 years ago, pathologists discovered that myocardial injury, particularly MI, triggers a profound inflammatory response. The injured myocardium is infiltrated by leukocytes, and these recruited leukocytes clear apoptotic cells and debris through phagocytosis, resulting in myocardial scarring. The properties of injured leukocytes and the close association between these leukocytes and cardiomyocytes at the border of injured myocardial tissues suggest that a subset of blood-derived cells can adhere to viable cardiomyocytes and may exert cytotoxic effects to amplify myocardial injury [27]. Specific approaches targeting molecules involved in leukocyte activation, adhesion and extravasation have successfully attenuated ischemic injury [28]. The pathogenesis of heart failure following MI is intricately linked to the development of postinfarct ventricular remodeling. Structural, functional and geometric alterations in infarcted and noninfarcted myocardial segments lead to ventricular dilation, increased ventricular sphericity and cardiac dysfunction [29]. Myocardial remodeling and heart failure progression increase the frequency of arrhythmias and survival of MI patients with a poor prognosis [30]. The degree of postinfarct remodeling depends on the size of the infarct area and the quality of the cardiac repair. Researchers have questioned the notion that inflammatory signaling prolongs ischemic injury [31,32]; however, inflammatory pathways undoubtedly play crucial roles in the dilatation and fibrotic remodeling of the infarcted heart, thereby driving the cardiac output of the injured myocardium, a key event in the pathogenesis of exhaustion. The repair of damaged tissue depends on the regulation of inflammatory factor levels, and the process is accompanied by the activation of integrity-related mesenchymal stem cells in tissues [33]. In damaged tissue, hyperinflammation can further damage myocardial tissue or function [33].

### Crosstalk of autophagy and inflammation in myocardial injury

Inflammation is a protective response during which the immune system activates and recruits a large number of inflammatory cells for infiltration when the body is injured or invaded by pathogens. Autophagy can influence the systemic immune response and the inflammatory responses of specific cell types. Inflammatory responses are associated with various CVD processes, such as myocardial I/R injury, atherosclerosis, cardiac remodeling and heart failure. Inflammation occurs mainly through pathogen-related molecular patterns or injury-related molecular patterns to induce multiprotein signal transduction cascades that secrete pro-inflammatory cytokines and activate adaptive immune responses. Ligands of Toll-like and nucleotide oligomerization domain (NOD) receptors not only induce inflammatory responses but also induce autophagy. In addition, studies have found that autophagy regulates Toll-like receptor (TLR) signals to enhance inflammatory infiltration [34,35]. Thus, autophagy is closely related to the occurrence of inflammatory diseases. This section mainly describes the relationship between autophagy and the inflammatory response in CVDs.

#### Autophagy regulates different types of immune cells

**Figure 2:**
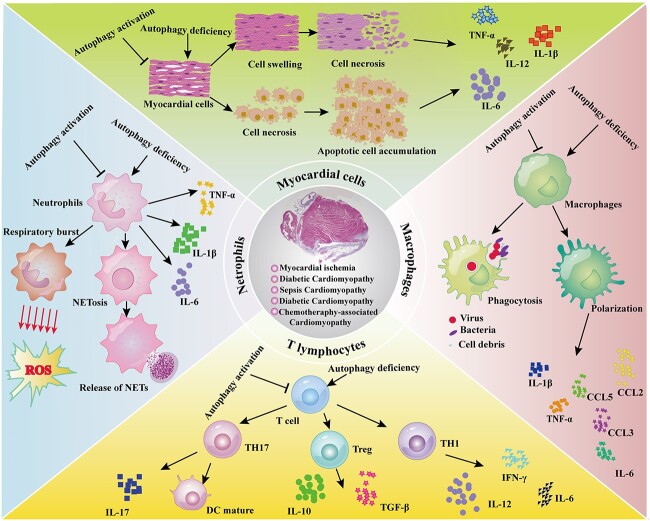
Autophagy regulates the inflammatory microenvironment of myocardial injury. *TNF-α*, Tumor necrosis factor α, *IL-1β*, interleukin 1β, *NETosis* neutrophil extracellular traposis, *ROS* reactive oxygen species, *NETs*, neutrophil extracellular traps, *CCL2* C-C motif chemokine ligand 2, *TH1* helper T cell 1, *Treg* regulatory T cell, *DC* dendritic cell, *IL-10* interleukin 10, *TGF-β* transforming growth factor-β, *IFN-γ* interferon-γ

When the myocardium is infected or in a state of ischemia and hypoxia, immune cells participate in the occurrence and development of myocardial inflammation. Autophagy protects cells from excessive inflammation via two mechanisms. Cells can effectively remove strong stimulants of inflammation, such as damaged organelles or disease-causing microorganisms. In addition, cells can protect other cells by inhibiting inflammatory complexes. Immune cells, including granulocytes, monocytes/macrophages and lymphocytes, play an important role in the inflammatory process of CVDs **(**Figure 2**)**. Investigating the inflammatory response of immune cells from the perspective of autophagy with the goal of reducing the inflammatory response in CVDs is of great significance to improve patient prognoses.

##### Autophagy regulates neutrophils

Neutrophils are multifunctional cells that play a central role in the innate immune system, which is the body’s first line of defense against the invasion of foreign pathogens. Neutrophils phagocytose and inactivate microorganisms and remove pathogens through the fusion of phagocytic cells and particles and the formation of autophagic lysosomes. The mechanism by which autophagy regulates neutrophil-mediated inflammatory injury is not clear at present. However, a large number of studies have shown that autophagy plays a key role in driving the inflammatory activity of neutrophils during myocardial injury [36,37].

Autophagy regulates ROS and cytokine production in neutrophils and is closely associated with myocardial injury. One of the mechanisms by which neutrophils regulate cardiac injury involves the production of a large number of ROS in a reduced nicotinamide adenine dinucleotide phosphate-dependent manner through a respiratory burst, which directly leads to tissue damage through the modification of proteins and lipids [38,39]. Studies have found that neutrophils from bone marrow-specific mice lacking ATG7 or ATG5 show reduced ROS production mediated by nicotinamide adenine dinucleotide phosphate oxidase [40], suggesting that autophagy defects reduce neutrophil-mediated myocardial injury. In addition, autophagy regulates neutrophil secretion of tumor necrosis factor (TNF)-α, interleukin (IL)-1β, IL-6 and other cytokines, aggravating the myocardial inflammatory microenvironment [41]. Autophagy inhibitors, such as 3-methyladenine, wortmannin, and bafilomycin A1, significantly reduced IL-1β secretion by neutrophils [42]. This finding suggests that autophagy may be associated with myocardial injury by regulating IL-1β secretion from neutrophils.

Neutrophil extracellular trap cell death is a form of programmed cell death. Activated neutrophils release depolymerized chromatin extracellularly when stimulated by certain cytokines, pathogens or compounds. The net structures are termed neutrophil extracellular traps (NETs). NETs not only engulf and kill invasive pathogens but also act as self-antigens, which leads to a variety of acute and chronic inflammatory reactions. Studies have found an increase in NETs in the culprit arteries of patients with acute MI, and these structures may play a central role in artery-blocking thrombosis by promoting fibrin deposition and the formation of the fibrin network [43]. The data suggest that NETs may be related to the occurrence of MI. Moreover, NETs are positively correlated with infarct size, adverse cardiac events and left ventricular dysfunction in patients with MI [44–46]. It has been reported [47] that NETosis requires autophagy and superoxide production, and inhibition of autophagy leads to cell death characterized by apoptosis rather than NETosis. Inhibition of mammalian target of rapamycin (mTOR) or autophagy activation by rapamycin can enhance the formation of NETs, whereas ATG5 deficiency can reduce the release of NETs [46,48], supporting the view that impaired neutrophil autophagy reduces NET release. Another study [49] found that autophagy-induced increases in NET levels were detected in neutrophils isolated from patients with sepsis. Neutrophils isolated from patients with sepsis showed autophagy dysregulation, and enhanced autophagy improved survival by increasing NET levels in a mouse model of sepsis [50]. Together, these studies strongly support the idea that NETs are closely related to autophagy pathways in myocardial injury.

##### Autophagy regulates macrophages

As an important component of innate immunity, macrophages are widely present in the tissue structure, including the heart, and play an important role in the development of the myocardium, tissue repair and remodeling, and the immune response. Autophagy in macrophages shows a wide range of functional characteristics that not only promote inflammation but also play a role in tissue repair [51]. Moreover, the characteristics of autophagy are different and complex during different stages of the same disease. Therefore, in-depth analysis of the diversity of macrophage function regulated by autophagy is of great significance for identifying new targets for disease intervention. This section summarizes the role of autophagy in cardiac macrophages during myocardial injury.

Coronary artery occlusion leads to ischemic injury of cardiomyocytes. Macrophages not only cause an intense inflammatory response and ventricular remodeling but are also necessary for inflammatory regression and cardiac repair. Myocardial injury induces the production and secretion of pro-inflammatory cytokines and chemokines by stationary macrophages, which trigger the metastasis of myeloid cells to the infarct site. The inflammatory response induced by myocardial injury plays an important role in myocardial healing and scarring, whereas a continuous inflammatory response greatly promotes myocardial remodeling and leads to heart failure. Apoptosis is a mechanism of debris removal and inflammation suppression during tissue injury and is closely related to macrophages. Metformin (Met) is a widely used hypoglycemic agent and has a significant cardioprotective effect during ischemic myocardial injury, such as acute MI (AMI). A study found that Met improved cardiac hemodynamics (left ventricular systolic pressure, left ventricular end-diastolic pressure, and maximum and minimum rate of change in the pressure in the ventricle over time [+DP/DT and −DP/DT, respectively]) and reduced the MI area in AMI mice by regulating macrophage autophagy and inhibiting the NOD-, LRR- and pyrin domain-containing protein 3 (NRLP3) inflammatory response. These results suggest that Met protects against ischemic myocardium injury by reducing the inflammatory response mediated by the macrophage autophagy–ROS–NLRP3 axis [52].

Reperfusion injury refers to the pathological process of the progressive aggravation of tissue injury when the ischemic myocardium is restored to normal perfusion after partial or complete acute obstruction of the coronary artery. The immune response plays an important role in I/R injury. NLRP3 inflammasome activation, lysosomal dysfunction and impaired autophagic flux play critical roles in the pathophysiology of I/R. In recent years, it has been found that macrophage autophagy plays a key role in NLRP3 activation during I/R [53]. In macrophages, lysosomes are at the center of key cellular processes that activate inflammasomes [54,55]. Induced macrophage-specific overexpressed transcription factor EB (TFEB) is a major regulator of lysosomal biogenesis. Javaheri *et al*. [56] established a mouse model of I/R injury and found that mice with TFEB overexpression exhibited lower levels of remodeling, pro-inflammatory macrophage abundance and secretion of IL-1β compared with the control group. Moreover, this process did not require ATG5-dependent autophagy. This finding suggests that TFEB reprograms lysosomal lipid metabolism in macrophages to attenuate remodeling of myocardial injury, suggesting another mode in which lysosomal function influences inflammation.

##### Autophagy regulates T lymphocytes

T lymphocytes obtain inflammatory information through membrane receptors, antigen-specific receptors and soluble mediators, and each tissue subpopulation identifies the lesion site according to the information and adjusts the characteristics of lymphocytes themselves to adapt to the changes in the microenvironment [57]. Lymphocyte subsets, including CD4+ T cells, CD8+ cells and regulatory T cells (Tregs), play an important role in regulating the inflammatory response that mediates the repair of myocardial injury. T cells effectively respond to changes in the intracellular and extracellular environment through autophagy regulation. Autophagy disorders may induce a variety of T-cell-related diseases [58,59]. In view of the potential application prospects of the T-cell autophagy pathway in the treatment of various diseases, this section summarizes the recent research progress that has been made in understanding the role of T-cell autophagy in regulating the inflammatory microenvironment that is present during myocardial injury. The regulatory role of autophagy in T-cell function and the mechanism of autophagy abnormalities during the process of myocardial injury are also discussed.

CD4+ and CD8+ T lymphocytes are the most complex immune cell populations known to play an important role in ameliorating myocardial injury after pathogen activation in a specific cytokine environment. Cecal ligation puncture (CLP) is the standard animal model for studying sepsis. The continuous spread of cecal contents into the abdominal cavity leads to the entry of bacteria into the bloodstream, ultimately leading to systemic inflammatory response syndrome and multiple organ dysfunction syndrome. Sepsis cardiomyopathy is one of the most important organ injuries. Lin *et al*. established a mouse model of sepsis myocardial injury using the CLP method and found that the number of autophagic vacuoles and autophagic lysosomes of spleen CD4+ and CD8+ T cells decreased in T-cell-specific ATG7-knockout (KO) mice [60]. In addition, with the inhibition of Th1/Th2/Th17 cytokines produced by CD4+ T cells, the phagocytotic ability of macrophages decreased, and the ability of the spleen to clear bacteria was inhibited after sepsis. These results suggest that autophagy may protect against T lymphocyte apoptosis and immunosuppression induced by sepsis.

Tregs play a key role in inhibiting myocardial inflammation after MI and are considered a subset of lymphocytes with anti-inflammatory properties. Deletion of ATG5 or ATG7 autophagy genes can regulate the immune homeostasis of Treg cells and reduce the number of Tregs [61]. The number of Tregs decreases with ischemic injury, and the adoptive transfer and expansion of Tregs can effectively reduce the development of ischemic tissue injury and promote repair [62]. Xia *et al*. [63] established a MI, I/R damage and frozen heart injury model in mice. In this model, Tregs were more highly enriched in the myocardium compared with the heart-draining lymph nodes. These results suggest that autophagy may participate in the regulation of myocardial injury by regulating Tregs.

#### Regulation of autophagy in injured nonimmune cells

Vascular endothelial cells constitute the continuous inner wall of the lumen of the cardiovascular system, which is a naturally formed blood vessel and a key node for maintaining homeostasis. Structural and functional injury of endothelial cells is the initial stage and an important marker of myocardial injury. The process of injury includes cell swelling, microvascular embolism and eventual rupture of microvessels, leading to cell apoptosis or the extravasation of contents, which is an inflammatory response that results in the deterioration of the myocardial microenvironment. In most cases of myocardial injury, autophagy activation can enhance endothelial cell activity, protect cells and maintain the homeostasis of the tissue environment. However, in some states, excessive autophagy destroys the protective barrier of endothelial cells, causes oxidative damage and ultimately promotes apoptosis [56]. Damaged microvessels cannot supply sufficient levels of oxygen, causing cardiomyocytes to swell and rupture and the subsequent worsening of the myocardial inflammatory microenvironment.

### Autophagy regulates damaged cardiomyocytes

MI, myocarditis, diabetes and other conditions can lead to myocardial cell damage, and autophagy plays an important role in regulating the inflammatory microenvironment of damaged myocardial cells. By regulating the autophagy-inflammation-related pathway of damaged cardiomyocytes, inflammatory cell infiltration and the levels of inflammatory factors can be reduced, alleviating cardiac injury [64–66].

NLRP3 is a typical inflammasome and plays an important role in the development of myocardial injury. Autophagy of cardiomyocytes can reduce the inflammatory response by eliminating active inflammatory bodies and stimuli with indirect inflammatory effects, and the accumulation of cellular metabolites and damaged organelles is closely related to the pathogenesis of myocardial injury [67–69]. Studies have shown that serine protease inhibitors (vaspin) can reduce myocardial injury by enhancing autophagy and inhibiting NLRP3 inflammasome activation [70]. The inhibitory effect of vaspin on NLRP3 inflammasome activation may depend on the upregulation of autophagy and ROS inhibition. Another study found that Met inhibits NLRP3 inflammation by activating adenosine monophosphate-activated protein kinase (AMPK)/mTOR autophagy-related pathways in dilated cardiomyopathy [71]. In a H9c2 cardiomyocyte hypoxia/reoxygenation model, thrombin aggravated hypoxia/reoxygenation damage in cardiomyocytes by activating the SIRT1-mediated autophagy pathway, which was reflected in the enhanced secretion of inflammatory factors, oxidative stress and decreased cell viability [72]. The inhibition of SIRT1 can improve these adverse effects. Viral myocarditis is a virus-induced local or diffuse, acute and chronic inflammatory disease of the myocardium [73], of which Coxsackie virus B3 is one of the major causes [74]. Rapamycin can be used to treat parasitic infections [75]. No changes in circulation or cardiac parasitemia were noted, whereas the cardiac inflammatory response was downregulated. Among them, rapamycin-treated infected mice exhibited preserved cardiac electrical function and reduced levels of cardiac damage, myocarditis and tissue pro-inflammatory cytokines interferon-γ, TNF-α and IL-6. These findings suggest that autophagy potentially played a role in maintaining cellular homeostasis and regulating inflammatory responses to ameliorate myocardial injury.

Aging is also an important factor in myocardial injury, and autophagy plays an important role in age-related cardiomyopathy. Cardiac senescent cardiomyopathy in mammals is characterized by myocardial hypertrophy and fibrosis, with a tendency toward myocardial cell apoptosis and autophagy [76]. The study investigated increased numbers of inflammatory cells in the hearts of older mice. A large number of autophagic vacuoles and lymphocyte clusters were present around the blood vessels that were not observed in the hearts of younger mice. In addition, the hearts of aged mice showed higher levels of Beclin-1 and LC3II protein expression, which was consistent with the induction of the autophagy pathway. However, other studies have found no difference in the expression of autophagy genes between the hearts of older and younger individuals, suggesting that the effects involve protein expression and occur after transcription.

### Regulation of autophagy in injured endothelial cells

Vascular endothelial cells are an important part of the cardiovascular system and their integrity underlies the ability of the cardiovascular system to exert homeostatic regulatory mechanisms. In addition, the secretion function of endothelial cells also plays an important role in the regulation of CVDs. Vascular endothelial dysfunction is the key and initial stage of myocardial injury, and the regulation of autophagy is closely related to a variety of vascular lesions caused by endothelial dysfunction. Endothelial cells can secrete a variety of vasoactive substances, which can be divided into two categories according to their regulatory functions on vascular smooth muscle. The first category comprises vasodilators, including nitric oxide (NO), endothelial-derived hyperpolarizing factor and prostacyclin. The other category includes vascular constrictors, such as endothelium-derived contracting factor, prostaglandin E2 and thromboxane A2.

Decreased endothelial nitric oxide synthase (eNOS) function leads to vascular motor dysfunction in patients with congestive heart failure and many other pathologic syndromes, such as left ventricular remodeling and dysfunction. Although the changes in cardiac function caused by NO are complex, numerous studies in recent years have demonstrated that the high expression of eNOS in both vascular endothelial cells and the myocardium can improve left ventricular function after MI [77,78]. eNOS participates in the protective role of ischemia postadaptation [79]. Ischemic postconditioning (IPostC) increased the formation of autophagosomes and upregulated the phosphorylation levels of eNOS and AMPK in models of I/R mice and H9c2 cells [80]. Inhibition of eNOS counteracts the improvement of autophagy by IPostC. This finding suggests that IPostC protects the heart from I/R injury in part by promoting AMPK/eNOS-mediated autophagy and is involved in the development of I/R. Abnormal mitochondrial fusion and division of endothelial cells are involved in the occurrence and development of many CVDs and are closely related to heart disease induced by I/R injury. Urolithin A (UA), a mitochondrial autophagy inducer, can reduce mitochondrial oxidative stress and stabilize mitochondrial membrane potential in endothelial cells to preserve mitochondrial function, upregulate cyclin D and E to maintain endothelial cell viability and improve endothelial cell proliferation. UA inhibits mitochondrial fission, restores mitochondrial fusion and reduces the proportion of mitochondrial fragments in endothelial cells. UA enhances mitochondrial biogenesis in endothelial cells by upregulating Sirtuin 3 and peroxisome proliferator-activated receptor γ coactivator 1-α [81]. These results suggest that activation of mitochondrial autophagy may alleviate cardiac microvascular injury induced by hypoxia/reoxygenation by improving mitochondrial quality control, cell viability and proliferation. In endothelial cells, autophagy can upregulate angiogenic activity and contribute to the repair of damaged endothelial cells by inducing prolonged hypoxia [82]. Mouse experiments also confirmed that autophagy activation can stimulate cell proliferation and regeneration near ischemic focal points.

### Autophagy affects the repair of damaged myocardium by regulating the inflammatory microenvironment

Inflammation is the body’s protective response to infection by pathogenic microorganisms or tissue damage. Inflammation occurs mainly through the innate immune system using pattern recognition receptors. Toll-like and Nod-like receptors recognize exogenous or endogenous ligands and are activated, and a multiprotein signal transduction cascade is subsequently induced to promote the secretion of pro-inflammatory cytokines. Inflammatory responses are associated with myocardial ischemia, reperfusion injury, septic cardiomyopathy, diabetic cardiomyopathy and heart failure. When myocardial injury occurs, organelles, microorganisms and a large number of damaged substances accumulate in cells **(**Figure 1**)**. Autophagy regulates the inflammatory microenvironment and inhibits the inflammatory response through the clearance of cellular debris [83]. However, autophagy can promote the occurrence of the inflammatory response [84]. Therefore, autophagy has a bidirectional effect and methods for regulating the inflammatory microenvironment during myocardial injury have not been determined. However, it is well known that autophagy and inflammation are closely associated with myocardial injury.

#### Myocardial ischemia

Myocardial ischemia is the pathological state of coronary artery lumen occlusion or stenosis in coronary heart disease, which is characterized by limited blood supply to the heart. The cardiac damage caused by acute MI is widely acknowledged to be caused by ischemic and I/R injuries, resulting in detrimental effects on cardiomyocytes. During the state of myocardial ischemia, the lack of raw materials leads to a decrease in ATP synthesis, which induces autophagy to clear apoptotic cardiomyocytes, misfolded proteins and necrotic mitochondria and regulates the myocardial inflammatory microenvironment [85]. Protecting cardiomyocytes is a vital strategy for the treatment of acute MI. The infiltration of immune cells has been observed in border areas after acute MI, indicating suppression of the inflammatory reaction in ischemic cardiac regions [86].

It was found that inflammatory factors and autophagy signals were strongest during the first week and apoptotic signals peaked during the second week after ligation of the left coronary artery, and the increased level persisted until the fourth week [87]. The application of a TNF-α inhibitor significantly inhibited autophagy and promoted muscle cell apoptosis in the boundary region. These results suggest that the inflammatory response may play a protective role in early MI by stimulating autophagy in myocardial cells. Myocardial-associated transcription factor A (MRTF-A) exerts an inhibitory effect on MI. Zhong *et al*. found that MRTF-A reduced the activity of the NLRP3 inflammasome and significantly increased the expression of autophagy proteins in ischemic myocardial tissue. Lipopolysaccharide and 3-methyladenine (3-MA) abrogated the protective effect of MRTF-A. Overexpression of MRTF-A and SIRT1 effectively reduced myocardial ischemia injury. This outcome was related to a decrease in inflammatory cytokine levels and an increase in autophagy-related protein levels. The inhibition of SIRT1 activity partially suppressed the cardiac protective effect induced by MRTF-A [88]. Superoxide dismutase 1 (SOD1)-KO mice showed excessive oxidative stress after AMI, which was caused by increased apoptosis of ischemic cardiomyocytes and an inflammatory response. In contrast, enhanced autophagy played a protective role. SOD1-KO mice had more severe myocardial inflammation after AMI than wild-type mice [89]. Vitamin D deficiency is associated with AMI [89]. A study found that vitamin D3 treatment enhanced the expression of LC3II and Beclin-1, reduced levels of inflammatory cell infiltration and the MI size in AMI mice, and decreased levels of inflammatory factors and MI markers, significantly alleviating AMI-induced myocardial cell apoptosis. Moreover, Bcl-2 upregulates or downregulates cysteine aspartic acid specific protease 3 (caspase-3), caspase-9 and Bax expression. In addition, vitamin D3 enhanced the inhibition of PI3K, P-Akt and P-mTOR expression induced by AMI [65]. The above experiments suggest that the pathway can promote autophagy in AMI-injured myocardium, protect against myocardial injury, inhibit the inflammatory response and improve the myocardial microenvironment.

Autophagy is closely related to the regulation of the inflammatory response to reperfusion injury after myocardial ischemia. Reperfusion injury is mainly due to acute injury caused by oxidative stress, which leads to ROS production after cardiomyocytes restore blood perfusion, and autophagy can reduce oxidative stress [90]. However, during I/R, excessive autophagy damages the myocardium [91]. ATP requirements are basically satisfied during I/R, and the main reason for autophagy activation is excessive oxidative-stress-induced ROS generation [91]. At present, the effect of autophagy on myocardial injury remains unclear and further exploration is needed.

Overall, the inflammatory environment promotes cell death during ischemia, whereas autophagy controls inflammation and protects myocardial function by inhibiting inflammasome activation. However, prolonged excessive autophagy may lead to the opposite effect by damaging cardiomyocytes. Autophagy and the myocardial inflammatory environment jointly regulate the entire process of myocardial ischemia.

#### Sepsis cardiomyopathy

Sepsis is a systemic inflammatory syndrome caused by infection that further develops into multiple organ dysfunction syndrome. The heart is one of the most vulnerable target organs. Many patients with severe sepsis have a decreased LVEF [92]. Improving the myocardial inflammatory microenvironment may represent a bottleneck in the treatment of sepsis.

In a mouse model of CLP sepsis, Hsieh *et al*. [93] reported that the colocalization of LC3 and lysosomal-associated membrane protein 1 decreased despite the elevation of LC3-II levels. Electron microscopy confirmed that autophagic flow was blocked during the late stage of sepsis, which manifested as an increase in the formation of autophagosomes in the left ventricle. However, reductions in their fusion with lysosomes hindered the degradation of autophagosomes, and the aggregation of autophagosomes promoted cardiac dysfunction and exacerbated septic cardiomyopathy. Busch *et al*. [94] established a sepsis-induced coagulopathy (SIC) model by CLP surgery and found that genes related to NF-κB signal transduction, autophagy and lysosomal protein degradation were enriched in the hearts of sepsis wild-type mice but not in sepsis NLRP3-KO mice. Further studies showed that IL-1β activated NF-κB and its target genes, resulting in myocyte myosin protein atrophy and reduction, which was accompanied by increased autophagy gene expression. Activation of the NLRP3 inflammasome induces cleavage of caspase-1 and IL-1β precursors into mature forms and their release, inducing downstream immune signaling responses. Autophagy can clear NLRP3 inflammasome activators, such as intracellular blockers, reducing the inflammatory response [95].

Mitochondria account for ~30% of the volume of myocardial cells. When insufficiencies in autophagy result in failure to clear damaged mitochondria, they accumulate in the cell and cause oxidative stress. Excessive ROS can activate downstream pathways to produce cascading inflammatory effects and worsen the inflammatory microenvironment. Mitochondria exist in a dynamic equilibrium state in which slightly damaged mitochondria can be repaired and complementarily fused with other damaged mitochondria into new mitochondria. Mitochondria that cannot be repaired are degraded by lysosomes [96]. During the pathological process of SIC, excessive ROS lead to oxidative stress and mitochondrial DNA damage as well as impaired mitochondrial protein synthesis and respiratory function. If damaged mitochondria are cleared by autophagy, mitochondrial biosynthesis is activated, which can alleviate myocardial injury caused by inflammation [97]. The process of septic cardiomyopathy is always accompanied by inflammation. Local pathological sections often exhibit inflammatory cell infiltration and deterioration of the inflammatory microenvironment. The mitochondrial structure and function of myocardial cells are damaged and mitochondrial autophagy is insufficient to clear damaged mitochondria. These damaged mitochondria accumulate in the cell and cause oxidative stress, induce the production of a large number of ROS and promote the inflammatory cascade reaction [98]. A large number of cardiomyocytes with morphological characteristics similar to pyroptotic cells were observed in the SIC animal model, and the presence of these cells was closely related to the inflammatory microenvironment. Autophagy can improve the inflammatory microenvironment by removing damaged DNA fragments, broken cell membranes, swollen organelles and cytoplasm.

#### Diabetic cardiomyopathy

Diabetic cardiomyopathy is one of the most important causes of death in patients with diabetes mellitus. Its pathological features mainly include structural and functional damage, including myocardial cell metabolism disorder, insulin resistance, oxidative stress, inflammatory response and neuroendocrine system disorders. The pathogenesis of diabetic cardiomyopathy myocardial injury remains unclear, but among many factors, the inflammatory response may play the most important role in promoting diabetic cardiomyopathy progression [99].

Typical autophagy is inhibited in type 1 diabetic hearts, and the reduction in autophagy is an adaptive change in type 1 diabetes that has a certain protective effect on cardiomyocytes [100]. Diabetes-induced heart injury was significantly weakened in Beclin 1- and ATG16-deficient diabetic mouse models [101]. These mice exhibited improved heart function and reduced levels of oxidative stress, interstitial fibrosis and myocardial cell apoptosis. In contrast, diabetic cardiac damage dose-dependently exacerbated Beclin 1 overexpression. These results suggest that reduced autophagy may represent an adaptive response to limit cardiac dysfunction in type 1 diabetes, possibly through upregulation of selective autophagy. Fenofibrate (FF) is a peroxisome proliferator-activated receptor α agonist that has reduced lipid levels in the clinic, and 3-MA or sirtinol has eliminated the preventive effect of FF on high-glucose production. These results suggest that FF may prevent the myocardial inflammatory response and dysfunction induced by type 1 diabetes by increasing FGF21 levels, which may upregulate SIRT1-mediated autophagy.

In type 2 diabetes induced by a high-fat diet, increased activation of typical autophagy has a protective effect on the myocardium. However, in type 2 diabetes induced by fructose and milk fat, increased activation of typical autophagy may aggravate myocardial injury [100]. One study observed increased expression of the cardiac autophagy marker LC3B-II and its mediator Beclin-1 and decreased expression of P62 in patients with type 2 diabetes. P62 was integrated into autophagosomes for effective degradation and promoted significant activation of apoptotic caspase-3. These results suggest that increased autophagy activity occurs in type 2 diabetic hearts.

#### Chemotherapy-associated cardiomyopathy

Cardiovascular toxicity caused by chemotherapy drugs has been increasingly recognized as an important factor affecting the survival and prognosis of cancer patients. The cardiotoxicity of anthracyclines is progressive and irreversible, with most symptoms appearing within 1 year of chemotherapy. During the process of anthracene cardiac injury, autophagy was found to be ‘unbalanced’ and unable to clear damaged apoptotic cells and damaged organelles, and a large amount of ROS accumulated, which induced an inflammatory cascade and aggravated heart failure [102–104].

The process of chemotherapy-related cardiomyopathy is always accompanied by inflammation, and both systemic and local inflammatory reactions occur. The systemic inflammatory response mainly occurs during the end stage of heart failure, whereas the local inflammatory response is a key factor in the process of heart failure that undergoes various adaptive compensatory mechanisms until decompensation and ultimately changes in myocardial structure, function and phenotype occur [105]. A recent study found that the IL-1 β concentration was positively associated with heart failure mortality. The risk of death was significantly increased at levels >49.1 pg/ml, whereas karazumab (IL-1 β-targeted) therapy reduced heart failure mortality [106], providing the first direct evidence that an anti-inflammatory drug can improve outcomes in patients with coronary heart disease. In addition, early heart failure was accompanied by elevated levels of inflammatory molecules and altered expression of genes involved in innate immunity, suggesting that inflammation and the innate immune system may represent an early response of cardiomyocytes to injury [107]. Ma *et al*. [108] established a mouse model of cardiomyopathy induced by peritoneal doxorubicin injection (10 mg/kg) and found that blocking the activity of TLR2 could reduce doxorubicin-induced cardiac insufficiency by 20% and inhibit myocardial fibrosis. In contrast, blocking TLR4 did not produce a similar phenomenon. Further studies showed that by disrupting the interaction between TLR2 and its endogenous ligand, the levels of inflammation and fibrosis in cardiomyocytes were reduced. However, inhibition of TLR4 exacerbates cardiac dysfunction and myocardial fibrosis by amplifying inflammation and inhibiting autophagy. These results suggest that autophagy interacts with TLR2 and TLR4 and plays different roles in chemotherapy-related cardiomyopathy.

### Autophagy modulators—strategic therapies for myocardial injury

Autophagy is critical to the degradation and disposal of damaged and dysfunctional organelles and protein aggregates and is a cellular ‘cleaning’ process. In addition to attention focused on its established fundamental role in maintaining normal cellular phenotype and function, interest in how targeted modulation of autophagy can prevent myocardial injury has increased.

The use of autophagy as a therapeutic modality has gained widespread support in recent years. Studies have found that autophagy regulators, including rapamycin [109], sulfaphenazole [110], UTP [111] and ranolazine [25] can reduce the myocardial infarct area, improve cardiac function and protect against myocardial ischemia. Rapamycin has been shown to reduce cardiomyocyte apoptosis and promote cardiomyocyte autophagy by modulating the crosstalk of mTOR and endoplasmic reticulum stress pathway components in the chronic heart failure context [112]. Rapamycin treatment of the myocardium significantly reduced the myocardial cell apoptosis rate, reduced the myocardial infarct area and enhanced cardiac function in mice after MI [113]. Met, one of the most widely used drugs for the treatment of type 2 diabetes, has also shown cardiovascular protective activity. Met enhanced autophagic flux and increased the regeneration of epicardium, endocardium and vascular endothelium [114]. Pretreatment with valsartan resulted in a significant reduction in myocardial infarct area in mice with I/R injury and induced autophagy in the hearts of rats after I/R injury. In contrast, the autophagy PI3K inhibitor 3-MA reduced the valsartan-induced enhancement of cardiac histology and reduction in infarct size in rats after I/R injury. Valsartan pretreatment induced autophagy through the AKT/mTOR/S6K pathway, independent of Beclin1 [115]. Chloroquine is included in the World Health Organization’s Essential Medicines List for the treatment of malaria and is an important autophagy inhibitor. An important study found that the use of chloroquine was associated with increased cardiac risk in patients with COVID-19 [116].

In conclusion, autophagy is considered as a potential therapeutic modality, however, some challenges remain. Specifically, whether autophagy is ‘beneficial’ or ‘detrimental’ during myocardial injury needs to be determined, as views differ. Myocardial injury is a dynamic process. The regulation of autophagy in myocardial injury is not static and needs to be maintained in a balanced state. It is important to understand the spatial–temporal change of autophagy during the whole process of myocardial injury, and autophagy regulation cannot be categorized as simply ‘good’ or ‘bad’. In most cases, clearly, the use of autophagy regulators in the treatment of myocardial injury shows considerable potential.

## Conclusions

Myocardial injury is a leading cause of morbidity and mortality worldwide. The cellular inflammatory response is closely related to the progression of myocardial injury. A complex relationship exists between autophagy and immune cells. Numerous studies have confirmed that autophagy is involved in regulating inflammatory responses, including the digestion of apoptotic necrotic cells, damage to organelles and macromolecules to inhibit excessive inflammatory responses to improve the microenvironment, and the promotion of inflammatory responses to repair tissues. The cell induces autophagy, clears inflammatory protein aggregates and downregulates the expression of pro-inflammatory cytokines produced during tissue damage to fight and improve the inflammatory response. Of note, the role of autophagy in regulating the inflammatory microenvironment of the myocardium is not absolute but is associated with changes in disease status. In-depth exploration of the functions and mechanisms of autophagy in human health and disease will provide new opportunities and approaches to develop methods for the prevention and treatment of inflammation and immune-related diseases.

## Abbreviations

AMI: Acute myocardial infarction; AMPK: Adenosine monophosphate-activated protein kinase; ATG: Autophagy-related gene; Caspase-1: cysteine aspartic acid specific protease; CLP: Cecal ligation puncture; CVD: Cardiovascular disease; eNOS: Endothelial nitric oxide synthase; FF: Fenofibrate; IL: Interleukin; IPostC: Ischemic postconditioning; I/R: Ischemia/reperfusion; KO: Knockout; LC3: Microtubule-associated protein 1 light chain 3; LAP: LC3-associated phagocytosis; 3-MA: 3-Methyladenine; Met: Metformin; MI: Myocardial infarction; MRTF-A: Myocardial-associated transcription factor A; mTORC1: Mammalian target of rapamycin complex 1; NETs: neutrophil extracellular traps; NRLP3: Nod-like receptor protein 3; NO: Nitric oxide; NOD: Nucleotide oligomerization domain; ROS: Reactive oxygen species; SIC: Sepsis-induced coagulopathy; SOD1: Superoxide dismutase 1; TNF: Tumor necrosis factor; TFEB: Transcription factor EB; TLR2: Toll-like receptor 2; Tregs: Regulatory T cells; UA: Urolithin A.

## Funding

This work was supported by the National Natural Science Foundation of China (82104495 and 82174161), Shenzhen Fundamental Research Program (No. SGDX20210823103804030), Science and Technology Development Fund, Macau SAR (No. 0025/2022/A1), University of Macau grants (No. MYRG2019-00129-ICMS), Macao Youth Scholars Program (AM2021023), Guangdong Basic and Applied Basic Research Foundation (2022A1515010395 and 2021A1515012573), Science and Technology Foundation of Guangzhou City (202102010257), State Key Laboratory of Dampness Syndrome of Chinese Medicine Research Foundation (SZ2021ZZ21 and SZ2022QN02), Scientific Research Projects of Guangdong Bureau of Traditional Chinese Medicine (Nos. 20212088), TCM Research Fund of Guangdong Provincial Hospital of Chinese Medicine (YN2020MS13) and The 2020 Guangdong Provincial Science and Technology Innovation Strategy Special Fund (Guangdong-Hong Kong-Macau Joint Lab, No. 2020B1212030006 and MY2022KF05).

## Authors’ contributions

CPL wrote the main article. YL drew the artwork. HC proofread the manuscript. XY was responsible for the insertion of the literature, and CJL, LW and JL were responsible for writing instructions and embellishing the text. All authors contributed to the article and approved the submitted version.

## Competing interests

None declared.

## References

[1] Roth GA, Mensah GA, Johnson CO, Addolorato G, Ammirati E, Baddour LM, et al. Global burden of cardiovascular diseases and risk factors, 1990-2019: update from the GBD 2019 study. J Am Coll Cardiol. 2020;76:2982–3021. 10.1016/j.jacc.2020.11.010. 33309175 PMC7755038

[2] Liu C, Chen J, Chen H, Zhang T, He D, Luo Q, et al. PCSK9 inhibition: from current advances to evolving future. Cell. 2022;11. 10.3390/cells11192972. PMC9562883 36230934

[3] Du J, Li Y, Zhao W. Autophagy and myocardial ischemia. Adv Exp Med Biol. 2020;1207:217–22. 10.1007/978-981-15-4272-5_15. 32671750

[4] Liu CP, Bayado N, He DY, Li J, Chen HQ, Li LM, et al. Therapeutic applications of extracellular vesicles for myocardial repair. Front Cardiovasc Med. 2021;8 ARTN 758050. 10.3389/fcvm.2021.758050. PMC8695616 34957249

[5] Liu C, Fan Z, He D, Chen H, Zhang S, Guo S, et al. Designer functional Nanomedicine for myocardial repair by regulating the inflammatory microenvironment. Pharmaceutics. 2022;14. 10.3390/pharmaceutics14040758. 35456592 PMC9025700

[6] Dillmann WH . Diabetic cardiomyopathy. Circ Res. 2019;124:1160–2. 10.1161/CIRCRESAHA.118.314665. 30973809 PMC6578576

[7] Higgins AY, O'Halloran TD, Chang JD. Chemotherapy-induced cardiomyopathy. Heart Fail Rev. 2015;20:721–30. 10.1007/s10741-015-9502-y. 26338137

[8] Lv X, Wang H. Pathophysiology of sepsis-induced myocardial dysfunction. Mil Med Res. 2016;3:30. 10.1186/s40779-016-0099-9. 27708836 PMC5037896

[9] Jefferies JL, Towbin JA. Dilated cardiomyopathy. Lancet. 2010;375:752–62. 10.1016/S0140-6736(09)62023-7. 20189027

[10] McComb S, Thiriot A, Akache B, Krishnan L, Stark F. Introduction to the immune system. Methods Mol Biol. 2019;2024:1–24. 10.1007/978-1-4939-9597-4_1. 31364040

[11] Sun K, Li YY, Jin J. A double-edged sword of immuno-microenvironment in cardiac homeostasis and injury repair. Signal Transduct Target Ther. 2021;6:79. 10.1038/s41392-020-00455-6. 33612829 PMC7897720

[12] Bento CF, Renna M, Ghislat G, Puri C, Ashkenazi A, Vicinanza M, et al. Mammalian autophagy: how does it work? Annu Rev Biochem. 2016;85:685–713. 10.1146/annurev-biochem-060815-014556. 26865532

[13] Liu C, Chen G, Chen Y, Dang Y, Nie G, Wu D, et al. Danlou tablets inhibit atherosclerosis in Apolipoprotein E-deficient mice by inducing macrophage autophagy: the role of the PI3K-Akt-mTOR pathway. Front Pharmacol. 2021;12:724670. 10.3389/fphar.2021.724670. 34566648 PMC8455997

[14] Gerada C, Ryan KM. Autophagy, the innate immune response and cancer. Mol Oncol. 2020;14:1913–29. 10.1002/1878-0261.12774. 32745353 PMC7463325

[15] Levine B, Mizushima N, Virgin HW. Autophagy in immunity and inflammation. Nature. 2011;469:323–35. 10.1038/nature09782. 21248839 PMC3131688

[16] Yang Z, Goronzy JJ, Weyand CM. Autophagy in autoimmune disease. J Mol Med (Berl). 2015;93:707–17. 10.1007/s00109-015-1297-8. 26054920 PMC4486076

[17] Mizushima N, Komatsu M. Autophagy: renovation of cells and tissues. Cell. 2011;147:728–41. 10.1016/j.cell.2011.10.026. 22078875

[18] Yang Z, Klionsky DJ. An overview of the molecular mechanism of autophagy. Curr Top Microbiol Immunol. 2009;335:1–32. 10.1007/978-3-642-00302-8_1. 19802558 PMC2832191

[19] Yu L, Chen Y, Tooze SA. Autophagy pathway: cellular and molecular mechanisms. Autophagy. 2018;14:207–15. 10.1080/15548627.2017.1378838. 28933638 PMC5902171

[20] Heckmann BL, Green DR. LC3-associated phagocytosis at a glance. J Cell Sci. 2019;132. 10.1242/jcs.222984. PMC6432721 30787029

[21] Boya P, Reggiori F, Codogno P. Emerging regulation and functions of autophagy. Nat Cell Biol. 2013;15:713–20. 10.1038/ncb2788. 23817233 PMC7097732

[22] Amaravadi R, Kimmelman AC, White E. Recent insights into the function of autophagy in cancer. Genes Dev. 2016;30:1913–30. 10.1101/gad.287524.116. 27664235 PMC5066235

[23] Mizushima N, Levine B. Autophagy in human diseases. N Engl J Med. 2020;383:1564–76. 10.1056/NEJMra2022774. 33053285

[24] Tanida I . Autophagy basics. Microbiol Immunol. 2011;55:1–11. 10.1111/j.1348-0421.2010.00271.x. 21175768

[25] Hale SL, Kloner RA. Ranolazine treatment for myocardial infarction? Effects on the development of necrosis, left ventricular function and arrhythmias in experimental models. Cardiovasc Drugs Ther. 2014;28:469–75. 10.1007/s10557-014-6548-3. 25112450

[26] Chanson M, Derouette JP, Roth I, Foglia B, Scerri I, Dudez T, et al. Gap junctional communication in tissue inflammation and repair. BBA-Biomembranes. 2005;1711:197–207. 10.1016/j.bbamem.2004.10.005. 15955304

[27] Entman ML, Youker K, Shoji T, Kukielka G, Shappell SB, Taylor AA, et al. Neutrophil induced oxidative injury of cardiac myocytes. A compartmented system requiring CD11b/CD18-ICAM-1 adherence. J Clin Invest. 1992;90:1335–45. 10.1172/JCI115999. 1357003 PMC443178

[28] Kluever AK, Deindl E. Extracellular RNA, a potential drug target for alleviating atherosclerosis, ischemia/reperfusion injury and organ transplantation. Curr Pharm Biotechnol. 2018;19:1189–95. 10.2174/1389201020666190102150610. 30605053

[29] Cohn JN, Ferrari R, Sharpe N. Cardiac remodeling--concepts and clinical implications: a consensus paper from an international forum on cardiac remodeling. Behalf of an international forum on cardiac Remodeling. J Am Coll Cardiol. 2000;35:569–82. 10.1016/s0735-1097(99)00630-0. 10716457

[30] White HD, Norris RM, Brown MA, Brandt PW, Whitlock RM, Wild CJ. Left ventricular end-systolic volume as the major determinant of survival after recovery from myocardial infarction. Circulation. 1987;76:44–51. 10.1161/01.cir.76.1.44. 3594774

[31] Briaud SA, Ding ZM, Michael LH, Entman ML, Daniel S, Ballantyne CM. Leukocyte trafficking and myocardial reperfusion injury in ICAM-1/P-selectin-knockout mice. Am J Physiol Heart Circ Physiol. 2001;280:H60–7. 10.1152/ajpheart.2001.280.1.H60. 11123218

[32] Chen W, Saxena A, Li N, Sun J, Gupta A, Lee DW, et al. Endogenous IRAK-M attenuates postinfarction remodeling through effects on macrophages and fibroblasts. Arterioscler Thromb Vasc Biol. 2012;32:2598–608. 10.1161/ATVBAHA.112.300310. 22995519 PMC3510666

[33] Ong SB, Hernandez-Resendiz S, Crespo-Avilan GE, Mukhametshina RT, Kwek XY, Cabrera-Fuentes HA, et al. Inflammation following acute myocardial infarction: multiple players, dynamic roles, and novel therapeutic opportunities. Pharmacol Ther. 2018;186:73–87. 10.1016/j.pharmthera.2018.01.001. 29330085 PMC5981007

[34] Ma K, Guo J, Wang G, Ni Q, Liu X. Toll-like receptor 2-mediated autophagy promotes microglial cell death by modulating the microglial M1/M2 phenotype. Inflammation. 2020;43:701–11. 10.1007/s10753-019-01152-5. 31834572

[35] Li XM, Jung KE, Yim SH, Hong DK, Kim CD, Hong JY, et al. Autophagy suppresses toll-like receptor 3-mediated inflammatory reaction in human epidermal keratinocytes. Biomed Res Int. 2020;2020:4584626. 10.1155/2020/4584626. 32461989 PMC7222544

[36] Mihalache CC, Simon HU. Autophagy regulation in macrophages and neutrophils. Exp Cell Res. 2012;318:1187–92. 10.1016/j.yexcr.2011.12.021. 22245582

[37] Ye X, Zhou XJ, Zhang H. Exploring the role of autophagy-related gene 5 (ATG5) yields important insights into autophagy in autoimmune/autoinflammatory diseases. Front Immunol. 2018;9:2334. 10.3389/fimmu.2018.02334. 30386331 PMC6199349

[38] Amulic B, Cazalet C, Hayes GL, Metzler KD, Zychlinsky A. Neutrophil function: from mechanisms to disease. Annu Rev Immunol. 2012;30:459–89. 10.1146/annurev-immunol-020711-074942. 22224774

[39] Ma Y, Yabluchanskiy A, Lindsey ML. Neutrophil roles in left ventricular remodeling following myocardial infarction. Fibrogenesis Tissue Repair. 2013;6:11. 10.1186/1755-1536-6-11. 23731794 PMC3681584

[40] Bhattacharya A, Wei Q, Shin JN, Abdel Fattah E, Bonilla DL, Xiang Q, et al. Autophagy is required for neutrophil-mediated inflammation. Cell Rep. 2015;12:1731–9. 10.1016/j.celrep.2015.08.019. 26344765

[41] Harris J . Autophagy and cytokines. Cytokine. 2011;56:140–4. 10.1016/j.cyto.2011.08.022. 21889357

[42] Iula L, Keitelman IA, Sabbione F, Fuentes F, Guzman M, Galletti JG, et al. Autophagy mediates interleukin-1beta secretion in human neutrophils. Front Immunol. 2018;9:269. 10.3389/fimmu.2018.00269. 29515581 PMC5825906

[43] Fuchs TA, Brill A, Duerschmied D, Schatzberg D, Monestier M, Myers DD, Jr, et al. Extracellular DNA traps promote thrombosis. Proc Natl Acad Sci U S A. 2010;107:15880–5. 10.1073/pnas.1005743107. 20798043 PMC2936604

[44] Hofbauer TM, Mangold A, Scherz T, Seidl V, Panzenbock A, Ondracek AS, et al. Neutrophil extracellular traps and fibrocytes in ST-segment elevation myocardial infarction. Basic Res Cardiol. 2019;114:33. 10.1007/s00395-019-0740-3. 31312919 PMC6647191

[45] Liu J, Yang D, Wang X, Zhu Z, Wang T, Ma A, et al. Neutrophil extracellular traps and dsDNA predict outcomes among patients with ST-elevation myocardial infarction. Sci Rep. 2019;9:11599. 10.1038/s41598-019-47853-7. 31406121 PMC6690880

[46] Helseth R, Shetelig C, Andersen GO, Langseth MS, Limalanathan S, Opstad TB, et al. Neutrophil extracellular trap components associate with infarct size, ventricular function, and clinical outcome in STEMI. Mediat Inflamm. 2019;2019:7816491. 10.1155/2019/7816491. PMC6854936 31772506

[47] Remijsen Q, Vanden Berghe T, Wirawan E, Asselbergh B, Parthoens E, De Rycke R, et al. Neutrophil extracellular trap cell death requires both autophagy and superoxide generation. Cell Res. 2011;21:290–304. 10.1038/cr.2010.150. 21060338 PMC3193439

[48] Xu F, Zhang C, Zou Z, Fan EKY, Chen L, Li Y, et al. Aging-related Atg5 defect impairs neutrophil extracellular traps formation. Immunology. 2017;151:417–32. 10.1111/imm.12740. 28375544 PMC5506403

[49] Mao JY, Zhang JH, Cheng W, Chen JW, Cui N. Effects of neutrophil extracellular traps in patients with septic coagulopathy and their interaction with autophagy. Front Immunol. 2021;12:757041. 10.3389/fimmu.2021.757041. 34707618 PMC8542927

[50] Park SY, Shrestha S, Youn YJ, Kim JK, Kim SY, Kim HJ, et al. Autophagy primes neutrophils for neutrophil extracellular trap formation during sepsis. Am J Respir Crit Care Med. 2017;196:577–89. 10.1164/rccm.201603-0596OC. 28358992

[51] Wu MY, Lu JH. Autophagy and macrophage functions: inflammatory response and phagocytosis. Cell. 2019;9. 10.3390/cells9010070. PMC7016593 31892110

[52] Fei Q, Ma H, Zou J, Wang W, Zhu L, Deng H, et al. Metformin protects against ischaemic myocardial injury by alleviating autophagy-ROS-NLRP3-mediated inflammatory response in macrophages. J Mol Cell Cardiol. 2020;145:1–13. 10.1016/j.yjmcc.2020.05.016. 32470468

[53] Lv S, Liu H, Wang H. The interplay between autophagy and NLRP3 Inflammasome in ischemia/reperfusion injury. Int J Mol Sci. 2021;22. 10.3390/ijms22168773. 34445481 PMC8395601

[54] Xia Y, Liu N, Xie X, Bi G, Ba H, Li L, et al. The macrophage-specific V-ATPase subunit ATP6V0D2 restricts inflammasome activation and bacterial infection by facilitating autophagosome-lysosome fusion. Autophagy. 2019;15:960–75. 10.1080/15548627.2019.1569916. 30681394 PMC6526827

[55] Zhang Z, Yue P, Lu T, Wang Y, Wei Y, Wei X. Role of lysosomes in physiological activities, diseases, and therapy. J Hematol Oncol. 2021;14:79. 10.1186/s13045-021-01087-1. 33990205 PMC8120021

[56] Javaheri A, Bajpai G, Picataggi A, Mani S, Foroughi L, Evie H, et al. TFEB activation in macrophages attenuates postmyocardial infarction ventricular dysfunction independently of ATG5-mediated autophagy. JCI Insight. 2019;4. 10.1172/jci.insight.127312. PMC6948771 31672943

[57] Liu C, Wang Y, Li L, He D, Chi J, Li Q, et al. Engineered extracellular vesicles and their mimetics for cancer immunotherapy. J Control Release. 2022;349:679–98. 10.1016/j.jconrel.2022.05.062. 35878728

[58] Yang G, Song W, Postoak JL, Chen J, Martinez J, Zhang J, et al. Autophagy-related protein PIK3C3/VPS34 controls T cell metabolism and function. Autophagy. 2021;17:1193–204. 10.1080/15548627.2020.1752979. 32268825 PMC8143264

[59] Dowling SD, Macian F. Autophagy and T cell metabolism. Cancer Lett. 2018;419:20–6. 10.1016/j.canlet.2018.01.033. 29339212 PMC5937942

[60] Lin CW, Lo S, Hsu C, Hsieh CH, Chang YF, Hou BS, et al. T-cell autophagy deficiency increases mortality and suppresses immune responses after sepsis. PLoS One. 2014;9:e102066. 10.1371/journal.pone.0102066. 25029098 PMC4100769

[61] Wei J, Long L, Yang K, Guy C, Shrestha S, Chen Z, et al. Autophagy enforces functional integrity of regulatory T cells by coupling environmental cues and metabolic homeostasis. Nat Immunol. 2016;17:277–85. 10.1038/ni.3365. 26808230 PMC4755832

[62] Zhuang R, Feinberg MW. Regulatory T cells in ischemic cardiovascular injury and repair. J Mol Cell Cardiol. 2020;147:1–11. 10.1016/j.yjmcc.2020.08.004. 32777294

[63] Xia N, Lu Y, Gu M, Li N, Liu M, Jiao J, et al. A unique population of regulatory T cells in heart potentiates cardiac protection from myocardial infarction. Circulation. 2020;142:1956–73. 10.1161/CIRCULATIONAHA.120.046789. 32985264

[64] Wang Y, Lu J, Cheng W, Gao R, Yang L, Yang Z. FK506 protects heart function via increasing autophagy after myocardial infarction in mice. Biochem Biophys Res Commun. 2017;493:1296–303. 10.1016/j.bbrc.2017.09.155. 28965948

[65] Wei YX, Dong SM, Wang YY, Zhang P, Sun MY, Wei YX, et al. Autophagy participates in the protection role of 1,25-dihydroxyvitamin D3 in acute myocardial infarction via PI3K/AKT/mTOR pathway. Cell Biol Int. 2021;45:394–403. 10.1002/cbin.11495. 33146448

[66] Fang J, Wang J, Chen F, Xu Y, Zhang H, Wang Y. alpha7nAChR deletion aggravates myocardial infarction and enhances systemic inflammatory reaction via mTOR-Signaling-related autophagy. Inflammation. 2019;42:1190–202. 10.1007/s10753-019-00979-2. 30806956

[67] Li X, Li Z, Li B, Zhu X, Lai X. Klotho improves diabetic cardiomyopathy by suppressing the NLRP3 inflammasome pathway. Life Sci. 2019;234:116773. 10.1016/j.lfs.2019.116773. 31422095

[68] Peng M, Liu Y, Xu Y, Li L, Li Y, Yang H. Cathelicidin-WA ameliorates diabetic cardiomyopathy by inhibiting the NLRP3 inflammasome. Cell Cycle. 2021;20:2278–90. 10.1080/15384101.2021.1981631. 34585633 PMC8794502

[69] Zhang H, Chen X, Zong B, Yuan H, Wang Z, Wei Y, et al. Gypenosides improve diabetic cardiomyopathy by inhibiting ROS-mediated NLRP3 inflammasome activation. J Cell Mol Med. 2018;22:4437–48. 10.1111/jcmm.13743. 29993180 PMC6111804

[70] Li X, Ke X, Li Z, Li B. Vaspin prevents myocardial injury in rats model of diabetic cardiomyopathy by enhancing autophagy and inhibiting inflammation. Biochem Biophys Res Commun. 2019;514:1–8. 10.1016/j.bbrc.2019.04.110. 31014675

[71] Yang F, Qin Y, Wang Y, Meng S, Xian H, Che H, et al. Metformin inhibits the NLRP3 Inflammasome via AMPK/mTOR-dependent effects in diabetic cardiomyopathy. Int J Biol Sci. 2019;15:1010–9. 10.7150/ijbs.29680. 31182921 PMC6535781

[72] Wang X, Xu Y, Li L, Lu W. Thrombin aggravates hypoxia/Reoxygenation injury of Cardiomyocytes by activating an autophagy pathway-mediated by SIRT1. Med Sci Monit. 2021;27:e928480. 10.12659/MSM.928480. 33931577 PMC8098101

[73] Pollack A, Kontorovich AR, Fuster V, Dec GW. Viral myocarditis--diagnosis, treatment options, and current controversies. Nat Rev Cardiol. 2015;12:670–80. 10.1038/nrcardio.2015.108. 26194549

[74] Knowlton KU . CVB infection and mechanisms of viral cardiomyopathy. Curr Top Microbiol Immunol. 2008;323:315–35. 10.1007/978-3-540-75546-3_15. 18357777

[75] Duque TLA, Cascabulho CM, Oliveira GM, Henriques-Pons A, Menna-Barreto RFS. Rapamycin treatment reduces acute myocarditis induced by Trypanosoma cruzi infection. J Innate Immun. 2020;12:321–32. 10.1159/000504322. 31801138 PMC7383291

[76] Boyle AJ, Shih H, Hwang J, Ye J, Lee B, Zhang Y, et al. Cardiomyopathy of aging in the mammalian heart is characterized by myocardial hypertrophy, fibrosis and a predisposition towards cardiomyocyte apoptosis and autophagy. Exp Gerontol. 2011;46:549–59. 10.1016/j.exger.2011.02.010. 21377520 PMC3104129

[77] Fraccarollo D, Widder JD, Galuppo P, Thum T, Tsikas D, Hoffmann M, et al. Improvement in left ventricular remodeling by the endothelial nitric oxide synthase enhancer AVE9488 after experimental myocardial infarction. Circulation. 2008;118:818–27. 10.1161/CIRCULATIONAHA.107.717702. 18678774

[78] Liu CP, Yeh JL, Wu BN, Chai CY, Chen IJ, Lai WT. KMUP-3 attenuates ventricular remodelling after myocardial infarction through eNOS enhancement and restoration of MMP-9/TIMP-1 balance. Br J Pharmacol. 2011;162:126–35. 10.1111/j.1476-5381.2010.01024.x. 20840538 PMC3012411

[79] Jones SP, Greer JJ, Kakkar AK, Ware PD, Turnage RH, Hicks M, et al. Endothelial nitric oxide synthase overexpression attenuates myocardial reperfusion injury. Am J Physiol Heart Circ Physiol. 2004;286:H276–82. 10.1152/ajpheart.00129.2003. 12969888

[80] Shao J, Miao C, Geng Z, Gu M, Wu Y, Li Q. Effect of eNOS on ischemic Postconditioning-induced autophagy against ischemia/reperfusion injury in mice. Biomed Res Int. 2019;2019:5201014. 10.1155/2019/5201014. 30881990 PMC6387714

[81] Wu D, Ji H, Du W, Ren L, Qian G. Mitophagy alleviates ischemia/reperfusion-induced microvascular damage through improving mitochondrial quality control. Bioengineered. 2022;13:3596–607. 10.1080/21655979.2022.2027065. 35112987 PMC8973896

[82] Jeong IH, Bae WY, Choi JS, Jeong JW. Ischemia induces autophagy of endothelial cells and stimulates angiogenic effects in a hindlimb ischemia mouse model. Cell Death Dis. 2020;11:624. 10.1038/s41419-020-02849-4. 32796816 PMC7429831

[83] Bravo-San Pedro JM, Kroemer G, Galluzzi L. Autophagy and Mitophagy in cardiovascular disease. Circ Res. 2017;120:1812–24. 10.1161/CIRCRESAHA.117.311082. 28546358

[84] Liu T, Han S, Dai Q, Zheng J, Liu C, Li S, et al. IL-17A-mediated excessive autophagy aggravated neuronal ischemic injuries via Src-PP2B-mTOR pathway. Front Immunol. 2019;10:2952. 10.3389/fimmu.2019.02952. 31921197 PMC6933613

[85] He J, Liu D, Zhao L, Zhou D, Rong J, Zhang L, et al. Myocardial ischemia/reperfusion injury: mechanisms of injury and implications for management (review). Exp Ther Med. 2022;23:430. 10.3892/etm.2022.11357. 35607376 PMC9121204

[86] Tibaut M, Mekis D, Petrovic D. Pathophysiology of myocardial infarction and acute management strategies. Cardiovasc Hematol Agents Med Chem. 2017;14:150–9. 10.2174/1871525714666161216100553. 27993119

[87] Wang X, Guo Z, Ding Z, Mehta JL. Inflammation, autophagy, and apoptosis after myocardial infarction. J Am Heart Assoc. 2018;7. 10.1161/JAHA.117.008024. PMC6015297 29680826

[88] Zhong Z, Luo XY, Xiang P, Ji HH, Wu XD, Chong AG, et al. MRTF-A alleviates myocardial ischemia reperfusion injury by inhibiting the inflammatory response and inducing autophagy. Mol Cell Biochem. 2022. 10.1007/s11010-022-04510-4. 35829871

[89] Bai YD, Yang YR, Mu XP, Lin G, Wang YP, Jin S, et al. Hydrogen Sulfide alleviates acute myocardial ischemia injury by modulating autophagy and inflammation response under oxidative stress. Oxidative Med Cell Longev. 2018;2018:3402809. 10.1155/2018/3402809. PMC6093072 30154948

[90] Xiang M, Lu Y, Xin L, Gao J, Shang C, Jiang Z, et al. Role of oxidative stress in reperfusion following myocardial ischemia and its treatments. Oxidative Med Cell Longev. 2021;2021:6614009. 10.1155/2021/6614009. PMC8149218 34055195

[91] Li L, Tan J, Miao Y, Lei P, Zhang Q. ROS and Autophagy: interactions and molecular regulatory mechanisms. Cell Mol Neurobiol. 2015;35:615–21. 10.1007/s10571-015-0166-x. 25722131 PMC11486209

[92] Furian T, Aguiar C, Prado K, Ribeiro RV, Becker L, Martinelli N, et al. Ventricular dysfunction and dilation in severe sepsis and septic shock: relation to endothelial function and mortality. J Crit Care. 2012;27:319 e9–15. 10.1016/j.jcrc.2011.06.017. 21855287

[93] Hsieh CH, Pai PY, Hsueh HW, Yuan SS, Hsieh YC. Complete induction of autophagy is essential for cardioprotection in sepsis. Ann Surg. 2011;253:1190–200. 10.1097/SLA.0b013e318214b67e. 21412148

[94] Busch K, Kny M, Huang N, Klassert TE, Stock M, Hahn A, et al. Inhibition of the NLRP3/IL-1beta axis protects against sepsis-induced cardiomyopathy. J Cachexia Sarcopenia Muscle. 2021;12:1653–68. 10.1002/jcsm.12763. 34472725 PMC8718055

[95] Biasizzo M, Kopitar-Jerala N. Interplay between NLRP3 Inflammasome and autophagy. Front Immunol. 2020;11:591803. 10.3389/fimmu.2020.591803. 33163006 PMC7583715

[96] Wong YC, Kim S, Peng W, Krainc D. Regulation and function of mitochondria-lysosome membrane contact sites in cellular homeostasis. Trends Cell Biol. 2019;29:500–13. 10.1016/j.tcb.2019.02.004. 30898429 PMC8475646

[97] Wu B, Song H, Fan M, You F, Zhang L, Luo J, et al. Luteolin attenuates sepsisinduced myocardial injury by enhancing autophagy in mice. Int J Mol Med. 2020;45:1477–87. 10.3892/ijmm.2020.4536. 32323750 PMC7138288

[98] Pan P, Wang X, Liu D. The potential mechanism of mitochondrial dysfunction in septic cardiomyopathy. J Int Med Res. 2018;46:2157–69. 10.1177/0300060518765896. 29637807 PMC6023059

[99] Kaur N, Guan Y, Raja R, Ruiz-Velasco A, Liu W. Mechanisms and therapeutic prospects of diabetic cardiomyopathy through the inflammatory response. Front Physiol. 2021;12:694864. 10.3389/fphys.2021.694864. 34234695 PMC8257042

[100] Kanamori H, Takemura G, Goto K, Tsujimoto A, Mikami A, Ogino A, et al. Autophagic adaptations in diabetic cardiomyopathy differ between type 1 and type 2 diabetes. Autophagy. 2015;11:1146–60. 10.1080/15548627.2015.1051295. 26042865 PMC4590644

[101] Xu X, Kobayashi S, Chen K, Timm D, Volden P, Huang Y, et al. Diminished autophagy limits cardiac injury in mouse models of type 1 diabetes. J Biol Chem. 2013;288:18077–92. 10.1074/jbc.M113.474650. 23658055 PMC3689952

[102] Asnani A . Activating autophagy to prevent doxorubicin cardiomyopathy: the timing matters. Circ Res. 2021;129:801–3. 10.1161/CIRCRESAHA.121.320063. 34591656

[103] Wallace KB, Sardao VA, Oliveira PJ. Mitochondrial determinants of doxorubicin-induced cardiomyopathy. Circ Res. 2020;126:926–41. 10.1161/CIRCRESAHA.119.314681. 32213135 PMC7121924

[104] Bartlett JJ, Trivedi PC, Pulinilkunnil T. Autophagic dysregulation in doxorubicin cardiomyopathy. J Mol Cell Cardiol. 2017;104:1–8. 10.1016/j.yjmcc.2017.01.007. 28108310

[105] Piper SE, McDonagh TA. Chemotherapy-related cardiomyopathy. Eur Cardiol. 2015;10:19–24. 10.15420/ecr.2015.10.01.19. 30310418 PMC6159418

[106] Pascual-Figal DA, Bayes-Genis A, Asensio-Lopez MC, Hernandez-Vicente A, Garrido-Bravo I, Pastor-Perez F, et al. The Interleukin-1 Axis and risk of death in patients with acutely decompensated heart failure. J Am Coll Cardiol. 2019;73:1016–25. 10.1016/j.jacc.2018.11.054. 30846095

[107] Frantz S, Bauersachs J, Kelly RA. Innate immunity and the heart. Curr Pharm Des. 2005;11:1279–90. 10.2174/1381612053507512. 15853684

[108] Ma Y, Zhang X, Bao H, Mi S, Cai W, Yan H, et al. Toll-like receptor (TLR) 2 and TLR4 differentially regulate doxorubicin induced cardiomyopathy in mice. PLoS One. 2012;7:e40763. 10.1371/journal.pone.0040763. 22808256 PMC3396603

[109] Stahli BE, Klingenberg R, Heg D, Branca M, Manka R, Kapos I, et al. Mammalian target of rapamycin inhibition in patients with ST-segment elevation myocardial infarction. J Am Coll Cardiol. 2022;80:1802–14. 10.1016/j.jacc.2022.08.747. 36049557

[110] Khan M, Mohan IK, Kutala VK, Kotha SR, Parinandi NL, Hamlin RL, et al. Sulfaphenazole protects heart against ischemia-reperfusion injury and cardiac dysfunction by overexpression of iNOS, leading to enhancement of nitric oxide bioavailability and tissue oxygenation. Antioxid Redox Signal. 2009;11:725–38. 10.1089/ARS.2008.2155. 18855521 PMC2850300

[111] Cohen R, Shainberg A, Hochhauser E, Cheporko Y, Tobar A, Birk E, et al. UTP reduces infarct size and improves mice heart function after myocardial infarct via P2Y2 receptor. Biochem Pharmacol. 2011;82:1126–33. 10.1016/j.bcp.2011.07.094. 21839729

[112] Gao G, Chen W, Yan M, Liu J, Luo H, Wang C, et al. Rapamycin regulates the balance between cardiomyocyte apoptosis and autophagy in chronic heart failure by inhibiting mTOR signaling. Int J Mol Med. 2020;45:195–209. 10.3892/ijmm.2019.4407. 31746373 PMC6889932

[113] Sun B, Xu Y, Liu ZY, Meng WX, Yang H. Autophagy assuages myocardial infarction through Nrf2 signaling activation-mediated reactive oxygen species clear. Eur Rev Med Pharmacol Sci. 2020;24:7381–90. 10.26355/eurrev_202007_21906. 32706077

[114] Xie F, Xu S, Lu Y, Wong KF, Sun L, Hasan KMM, et al. Metformin accelerates zebrafish heart regeneration by inducing autophagy. NPJ Regen Med. 2021;6:62. 10.1038/s41536-021-00172-w. 34625572 PMC8501080

[115] Wu XQ, He LS, Cai Y, Zhang GP, He YL, Zhang ZJ, et al. Induction of autophagy contributes to the myocardial protection of valsartan against ischemia-reperfusion injury. Mol Med Rep. 2013;8:1824–30. 10.3892/mmr.2013.1708. 24084854

[116] Cohen IV, Makunts T, Moumedjian T, Issa MA, Abagyan R. Cardiac adverse events associated with chloroquine and hydroxychloroquine exposure in 20 years of drug safety surveillance reports. Sci Rep-Uk. 2020;10 ARTN 19199. 10.1038/s41598-020-76258-0. PMC7644696 33154498

